# Procaryotic Diversity and Hydrogenotrophic Methanogenesis in an Alkaline Spring (La Crouen, New Caledonia)

**DOI:** 10.3390/microorganisms9071360

**Published:** 2021-06-23

**Authors:** Marianne Quéméneur, Nan Mei, Christophe Monnin, Anne Postec, Laura Wils, Manon Bartoli, Sophie Guasco, Bernard Pelletier, Gael Erauso

**Affiliations:** 1 Aix Marseille Univ, Université de Toulon, CNRS, IRD, MIO, Marseille, France; meinankh@163.com (N.M.); anne.postec@mio.osupytheas.fr (A.P.); laura.wils@hotmail.fr (L.W.); manon.joseph@mio.osupytheas.fr (M.B.); sophie.guasco@mio.osupytheas.fr (S.G.); gael.erauso@mio.osupytheas.fr (G.E.); 2 GET UMR5563 (CNRS/UPS/IRD/CNES), Géosciences Environnement Toulouse, 14 Avenue Edouard Belin, 31400 Toulouse, France; christophe.monnin@get.omp.eu; 3 IRD, UMR Géoazur (UCA, CNRS, IRD, OCA), Centre IRD de Nouméa, BP A5, 98848 Nouméa, Nouvelle-Calédonie; bernard.pelletier@ird.fr

**Keywords:** archaea, bacteria, *Methanobacterium*, alkaline spring, microbial diversity, alkaliphiles, hydrogenotrophy, methanogenesis

## Abstract

(1) Background: The geothermal spring of La Crouen (New Caledonia) discharges warm (42 °C) alkaline water (pH~9) enriched in dissolved nitrogen with traces of methane, but its microbial diversity has not yet been studied. (2) Methods: Cultivation-dependent and -independent methods (e.g., Illumina sequencing and quantitative PCR based on 16S rRNA gene) were used to describe the prokaryotic diversity of this spring. (3) Results: Prokaryotes were mainly represented by *Proteobacteria* (57% on average), followed by *Cyanobacteria, Chlorofexi,* and *Candidatus* Gracilibacteria (GN02/BD1-5) (each > 5%). Both potential aerobes and anaerobes, as well as mesophilic and thermophilic microorganisms, were identified. Some of them had previously been detected in continental hyperalkaline springs found in serpentinizing environments (The Cedars, Samail, Voltri, and Zambales ophiolites). *Gammaproteobacteria*, *Ca.* Gracilibacteria and *Thermotogae* were significantly more abundant in spring water than in sediments. Potential chemolithotrophs mainly included beta- and gammaproteobacterial genera of sulfate-reducers (*Ca.* Desulfobacillus), methylotrophs (*Methyloversatilis*)*,* sulfur-oxidizers (*Thiofaba, Thiovirga*), or hydrogen-oxidizers (*Hydrogenophaga*). Methanogens (*Methanobacteriales* and *Methanosarcinales*) were the dominant *Archaea*, as found in serpentinization-driven and deep subsurface ecosystems. A novel alkaliphilic hydrogenotrophic methanogen (strain CAN) belonging to the genus *Methanobacterium* was isolated, suggesting that hydrogenotrophic methanogenesis occurs at La Crouen.

## 1. Introduction

Several thermal springs associated with low-temperature serpentinization can be found in the southeastern part of the main island of New Caledonia, either on land or in the southern lagoon [1,2,3,4]. At the northern end of the “Massif du Sud”, which is the main part of the New Caledonia ophiolite, several springs can be found near the city of Canala in a very fractured area at the transition between autochthonous sediments and the southern peridotite nappe [4]. Data reported for two of these springs showed that they discharge alkaline water (pH~9) at ~42 °C [3,5]. Deville and Prinzhofer [3] have shown that gases bubbling at the spring they studied in this area were composed of nitrogen (N_2_) with traces of methane (CH_4_), but no hydrogen (H_2_) was detected.

The geological environment and the chemical composition of the La Crouen waters differ from those of the hyperalkaline springs located down south in the Prony Bay. There, warm (up to 42 °C) high-pH (up to 11.2) hydrothermal fluid produced by the serpentinization of the ophiolite discharges in the lagoon at depths from shallow (<50 m bsl) to intertidal [1,2]. Mixing with ambient seawater leads to the formation of large (up to ten meters high) chimneys of brucite-carbonate, the most prominent being the Aiguille de Prony [1,2]. This water discharge is accompanied by N_2_-H_2_-CH_4_ gas bubbling at the springs [2,3]. The Prony Bay Hydrothermal Field (PBHF) bears similarities with the deep-sea Lost City hydrothermal field (LCHF) located at ~800 m bsl, off the Mid-Atlantic Ridge (30° N) [6]. Both submarine LCHF and PBHF shared a low archaeal diversity dominated by a few uncultured *Methanosarcinales* potentially involved in CH_4_ production and oxidation in anaerobic conditions [7,8,9,10,11,12]. In addition, several abundant and diverse bacterial phylotypes (with different potential metabolisms) like those of the PBHF are found in several continental serpentinizing sites worldwide (e.g., The Cedars, Samail Ophiolite) [10,11,12,13]. Several anaerobic alkaliphilic bacteria were isolated from the PBHF waters [14,15,16,17,18,19,20]. They were described as new species of *Alkaliphilus* [15,20] or a new genus (*Serpentinicella*) [19], all belonging to the same family *Clostridiaceae* (phylum *Firmicutes*). While the PBHF ecosystem has been given much attention, the microbial diversity of the New Caledonia on-land alkaline springs had not been studied before the work reported here.

Different abiotic or biotic processes are known to form CH_4_ in natural environments (e.g., abiotic thermal maturation of organic matter, Fischer–Tropsch and Sabatier reactions, and microbial methanogenesis) [21,22]. Microbial processes occurring in natural anoxic and extreme habitats (e.g., hot springs, submarine hydrothermal vents, subsurface) are mainly mediated by anaerobic methanogenic *Archaea* producing CH_4_ as a metabolic by-product of CO_2_ reduction, methyl-group reduction, or acetoclastic reaction. They thus contribute to the primary production when using CO_2_ or small organic molecules of abiotic origin [23,24]. Aerobic degradation of methylphosphonate by marine bacterioplankton has also been mentioned to contribute to CH_4_ production in the sea and ocean waters [25,26]. In continental hyperalkaline springs, molecular studies have recently evidenced active microbial CH_4_ production and oxidation in the serpentinite-hosted waters of the Voltri Ophiolitic springs (Italy) [27] and those of the Samail Ophiolite (Oman) [28]. Indeed, both anaerobic archaeal methanogens *Methanobacteriaceae* and aerobic bacterial methanotrophs *Methylococcaceae* were prevalent at these continental sites [27,28,29]. At the alkaline site of La Crouen (New Caledonia), Deville and Prinzhofer [3] have measured a low CH_4_ content (2.65−2.73%), with a δ^13^C signature of −39%, suggesting a possible thermogenic origin of CH_4._ However, the δ^13^C isotopic signature of CH_4_ cannot provide an unambiguous clue to its mode of formation because of the overlap between the various genetic fields in isotopic diagrams [3,30].

In this study, we used MiSeq Illumina sequencing of the 16S rRNA gene to study the composition of prokaryotic communities in the alkaline thermal spring at La Crouen (New Caledonia) and compared the dominant members with those of the alkaline serpentinite-hosted springs of other ecosystems worldwide. We also used quantitative PCR targeting the *mcrA* gene encoding the methyl-Coenzyme M reductase (the key enzyme catalyzing the last step of the methanogenesis) and anaerobic cultivation methods to explore the methanogens diversity and their CH_4_ production potential.

## 2. Materials and Methods

### 2.1. Study Site

**Figure 1:**
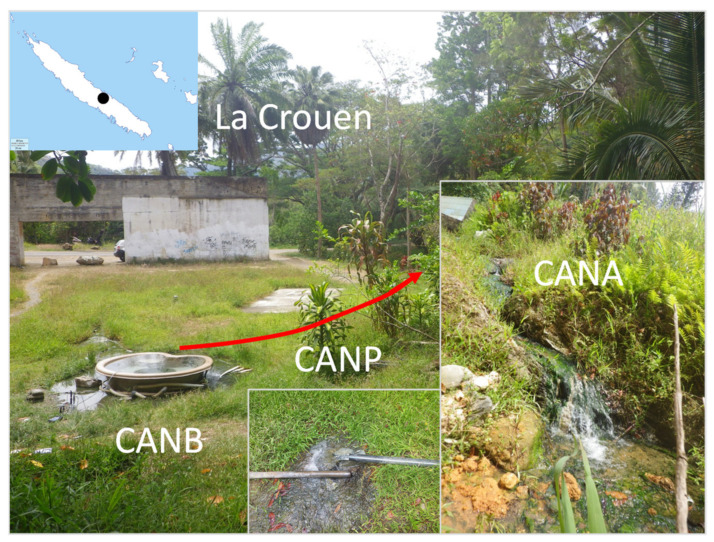
Location and photographs of the alkaline spring of La Crouen spring (New Caledonia). Photographs show the Scheme 100 mL glass bottles sealed with butyl stoppers. For molecular microbial community analysis, both waters and sediments were collected in duplicate at CANB, CANP, and CANA. Surface sediments were sampled with a sterile spatula and collected into sterile Falcon tubes. Water samples were collected in cleaned 4 L plastic bottles. Both water and sediment samples were stored in a portable icebox until arrival at the laboratory (about two hours after sampling). Two liters of water were filtered in duplicate through 0.2 μm pore-size Isopore polycarbonate membrane filters (Millipore). The filters and sediments were kept at −80 °C before DNA extraction. A sterile glass bottle was also filled in the field with the sediment slurry, then hermetically sealed to prevent oxidation, and stored at 4 °C before cultivation.

Several springs have been known about for a long time in the area of La Crouen, close to the town of Canala, approximately 165 km north of Nouméa, New Caledonia (see the map in [4]). The spring we have studied is located at La Crouen (Figure 1; 165° 53′ 20.6” E 21° 32′ 06.6” S), where it has been captured to feed a now derelict spa facility, founded in 1946 but abandoned for more than 30 years.

### 2.2. Sample Collection

Water and sediments samples were collected on 28 November 2014 at three locations at La Crouen spring: (i) the source bathtub (CANB), (ii) the outlet of a pipe next to a meter-wide pool below the source (CANP), and (ii) a small pool with green algae (CANA), approximately 3 m below CANP before its entry into the river (Figure 1). 

The oxidation–reduction potential (ORP), O_2_, pH, temperature, and conductivity were measured in situ using a WTW Multi 3420^®^ Multimeter with adequate probes at CANB, CANP, and CANA.

### 2.3. Chemical Analysis

All chemical analyses were carried out at the Geosciences Environnement Toulouse (GET) laboratory. Cation (Na+, K^+^, Ca^2+^, Mg^2+^, Ba^2+^, Sr^2+^, and Si^4+^) and total sulfur (reported as SO_4_) concentrations were measured using an ICP-OES Horiba Jobin Yvon Ultima2^®^. The dissolved inorganic carbon content (DIC) and the non-purgeable dissolved organic carbon content (NPOC) were measured with a Shimadzu^®^ analyzer. The analytical precisions for the ICP-OES and the carbon analyzer are 5% and 2%, respectively. The anion concentrations (Cl^−^, SO_4_^2−^, NO_3_^−^, NO_2_^−^, F^−^) were measured by ionic chromatography (Dionex ICS 2000^®^ liquid chromatographer).

Dissolved gas analysis was performed using a headspace equilibration method adapted from Magen et al. [31]. Briefly, a headspace representing about 10% of the vial volume (10 mL) was created in the collection bottle by water displacement with argon, then the bottle was manually shaken for 1 min and placed on a shaker for 1 h. The composition of the headspace gas was determined using a Shimadzu GC 8A gas chromatograph equipped with a thermal conductivity detector (GC/TCD) and a concentric column CTR1 (Alltech, Deerfield, IL, USA), as described by Mei et al. [17]. Argon was used as carrier gas at a flow rate of 60 mL/min; the temperature of the injector and the detector was fixed at 150 °C.

### 2.4. Methane Production in Anaerobic Enrichment Cultures

The potential for microbial CH_4_ production from La Crouen spring was evaluated through anaerobic enrichment cultures amended with methanogenic substrates (acetate, formate, and H_2_/CO_2_) of CANB sample as inoculum (displaying the lowest ORP value, i.e., −351 mV, required for methanogenesis ranging between −200 mV and −400 mV at pH 7 [32,33]). The culture medium contained (per liter of distilled water): 0.1 g KH_2_PO_4_, 0.1 g K_2_HPO_4_, 0.5 g NH_4_Cl, 0.5 g NaCl, 0.002 g FeSO_4_·7H_2_O, 0.1 g CaCl_2_·2H_2_O, 0.1 g MgSO_4_·7H_2_O, 2 g sodium formate, 1g sodium acetate, 0.1 g cysteine hydrochloride and 1 mL trace element solution SL-10 [34]. The initial pH was adjusted to 9 with NaOH. Then, the culture medium was boiled for 5 min and cooled to room temperature under a flow of O_2_-free N_2_ gas. The medium was dispensed into Hungate tubes, degassed under a flow of N_2_, and subsequently autoclaved (20 min, 120 °C). The following sterile solutions were injected in each tube: 0.1 mL of 2% Na_2_S·9H_2_O, 0.1 mL of 8% Na_2_CO_3_, 0.1 mL of 10 % tryptone and 0.1 mL of 10% yeast extract. Inoculation at 10% (*v*/*v*) was done with 0.5 mL of the CANB slurry sample. Each tube was supplemented by H_2_/CO_2_ gas mixture (80:20 *v*/*v*; at 2 bars). The suspensions were serially diluted in decimal steps using the same media (up to 10^−4^) and then incubated for one month at 37 °C under shaking (100 rpm). The Hungate technique for anaerobic cultivation was used throughout this study [35]. One hundred microliters of the headspace gas were periodically analyzed to determine its CH_4_ content (as indicated above). The presence of putative methanogens from CH_4_-producing enrichments was checked by observing cofactor-F420 autofluorescence in methanogenic cells using a Nikon Eclipse E600 equipped for epifluorescence.

### 2.5. Isolation and Identification of Methanogens

The CH_4_-producing culture of CANB with the highest dilution (10^−4^) was subcultured into the medium described above (without sodium acetate, sodium formate, yeast extract, and tryptone) with the addition of Balch vitamin solution [36], and H_2_/CO_2_ (80:20 *v*/*v*, 2 bars) as the only source of carbon. Hydrogenotrophic CH_4_-producing cultures were then purified by repeated use of the Hungate roll-tube method [35] with solid medium (1.6% *w*/*v* agar, Difco). Several colonies that had developed were picked and cultivated in minimum liquid medium with the addition of Balch vitamins and H_2_/CO_2_ (80:20 *v*/*v*, 2 bars). Microbial growth in cultures was monitored by measuring the increase in turbidity at 600 nm after insertion of Hungate tubes into the cuvette holder of a UV-visible spectrophotometer (Cary 50, Varian). Gas evolution in cultures was determined as indicated above. The purity of a selected isolate in culture, noted CAN, was checked after DNA extraction (as indicated below), followed by the amplification of the V3 hypervariable region of the 16S rRNA gene using the primer set 341F/518R for the Bacteria domain [37] to verify the absence of bacterial DNA amplification, then with the primer set A109F/1492R for *Archaea* domain [38,39] and then sequenced by GATC-Biotech (Konstanz, Germany). The 16S rRNA gene sequence of the CAN strain (KR349725) was compared with those present in the NCBI non-redundant database, using the BLASTn search tool [40]. A selection of representative homologous sequences was then aligned with CAN 16S rRNA gene sequence using MUSCLE [41], and phylogenetic trees were constructed and evaluated using the maximum-likelihood method [42] implemented in MEGA7 software [43].

### 2.6. DNA Extraction, PCR, and MiSeq Illumina Sequencing of 16S rRNA Genes from Sediment and Water Samples

DNA was extracted from duplicate sediment samples (CANBS1, CANBS2, CANPS1, CANPS2, CANAS1, CANAS2) and filters (CANBF1, CANBF2, CANPF1, CANPF2, CANAF1, CANAF2) following the protocol previously described by Quéméneur et al. [11]. The DNA concentrations were measured using a Qubit^®^ fluorometer (Invitrogen).

Abundance of *Bacteria* and *Archaea* was determined by quantitative PCR (qPCR) using respectively the primer sets 341F/518R [37] and 344F/519R [44,45]. The primer set used to quantify methanogens was ME3MF/ME2r’ targeting *mcrA* genes [46,47]. The quantitative PCR was performed in triplicate in a Bio-Rad CFX-96 real-time system (Bio-Rad). The qPCR assays protocol and gene copy number calculation have been previously described by Quéméneur et al. [11]. The abundance of targeted genes was reported as copy numbers per L for water or g for sediment.

Bacterial and archaeal 16S rRNA gene V4 hypervariable regions were amplified by PCR using the 515F/806R universal primer set [48], with a barcode on the forward primer, as previously described by Dowd et al., and were sequenced by the MiSeq Illumina platform of the Molecular Research Laboratory (Shallowater, TX, USA) [49]. Sequence data were processed using MR DNA analysis pipeline (MR DNA, Shallowater, TX, USA). In summary, sequences were joined, barcodes were removed, then short sequences (<150 bp) and sequences with ambiguous base calls were removed. Sequences were denoised, then Operational Taxonomic Units (OTUs) generated, and chimeras removed. OTUs were defined by clustering at 97% of similarity. Finally, OTUs were taxonomically classified using BLASTn against the NCBI non-redundant (NR) reference database. The 16S rRNA gene sequences of the dominant OTUs have been deposited in the Genbank database under the accession numbers MW801388-MW801436.

### 2.7. Statistical Analyses

All statistical analyses were performed using XLSTAT 2020.5.1 (Microsoft Excel add-in program; Addinsoft, Paris, France). The alpha diversity was assessed by calculating Shannon [50] and Simpson [51] indices. Non-parametric Kruskal–Wallis test, followed by Dunn’s test with Bonferroni correction, was used to evaluate differences in the alpha diversity indices and in the relative abundance of microbial taxa (classes/phyla and dominant OTUs) between waters (W) and sediments (S). *p* values < 0.05 are statistically significant. *p* values < 0.05 indicate that there are differences between the two groups (S and W). The abundance of the dominant OTUs in the studied samples was also visualized by heatmap. 

## 3. Results

### 3.1. Chemical Composition of the Waters

In situ measurements along with the outflow of La Crouen spring (from source pool CANB to CANA stream, ~10 m) showed an increase in both dissolved oxygen (O_2_) levels (from 0.7 to 4.9 mg/L) and ORP values (−351 to −260 mV, ref. Ag/AgCl) with increasing distance from the spring outlet, while temperature and pH values were similar along the water outflow (Table 1). The water in the bathtub (CANBW) had a temperature of 41 °C, an alkaline pH ~9, and low dissolved O_2_ (0.7 mg/L), and ORP (−351 mV). The chloride (0.24 mM), sulfate (0.16 mM) and dissolved organic carbon (NPOC, 0.05 mM) (Table S1) were low. The dissolved H_2_ and CH_4_ concentrations of CANBW waters were below detection limit (<0.1% vol). N_2_ was the main dissolved gas (about 363 µM) in our samples (Table S1).

### 3.2. Abundance and Diversity of Prokaryotic Communities

Real-time qPCR assays and MiSeq Illumina sequencing of the amplicon of the V4 region of the 16S rRNA gene were performed on 12 different samples (6 of waters and 6 of sediments) collected along with the water outflow of La Crouen spring. The suffixes W and S at the end of the sampling site name in Table 1 designate, respectively, the water and sediment samples collected from each site (CANB, CANP, and CANA). No significant difference in the Simpson diversity indices (*D*) was observed between the water samples (*D* = 0.94 ± 0.02 on average) and the sediments (on average *D* = 0.96 ± 0.02; *p >* 0.05) (Table 1). On the contrary, the Shannon diversity indices (*H*) in the sediment samples (*H* = 5.0 ± 0.4 in average) was significantly higher than that in the waters (on average *H* = 4.1 ± 0.4; *p =* 0.025), indicating a high frequency of low abundance phylotypes in the sediments. No significant difference in both *D* and *H* indices was observed along the path of water outflow. However, the highest *D* and *H* values were observed in the source CANB samples, suggesting a high microbial diversity in these samples.

The qPCR experiments revealed that the bacterial 16S rRNA gene abundance ranged between 3.04 × 10^7^ (CANPW) and 1.06 × 10^9^ (CANAW) copies/L of water and between 2.42 × 10^9^ (CANPS) and 6.97 × 10^9^ (CANBS) copies/g of sediment. The archaeal 16S rRNA gene abundance varied from 1.92 × 10^6^ (CANPW) to 1.63 × 10^7^ (CANAW) copies per L of water and gradually increased from 5.38 × 10^7^ (CANBS) to 8.69 × 10^7^ (CANAS) copies per g of sediments (Table 1). The bacterial 16S rRNA genes were approximately 100 times more abundant than archaeal 16S rRNA genes in sediments, while they were 34 times on average more abundant than archaeal genes in waters. The *mcrA* genes (methanoarchaea functional gene markers) were detected in all samples but were more abundant in sediment (e.g., 1.95 × 10^7^ copies per g in a CANBS sample), where methanogens represented up to 68% of the archaeal community abundance.

### 3.3. Composition and Distribution of Prokaryotic Communities

**Figure 2:**
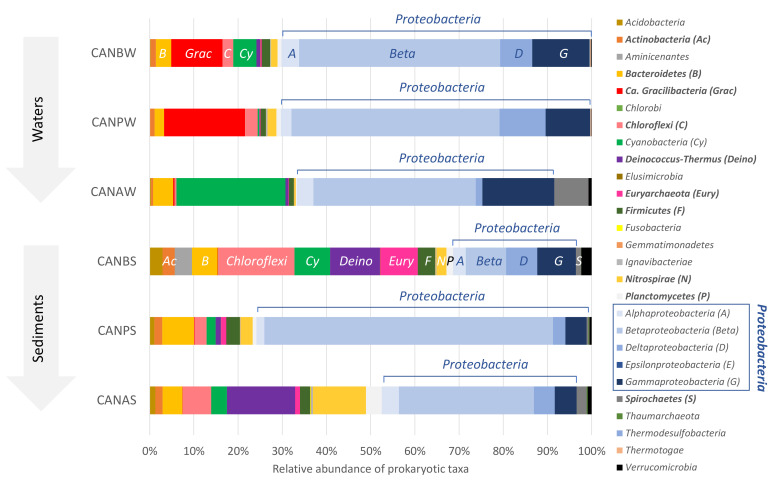
Distribution of the prokaryotic taxa in the waters and sediment samples along the water outflow of La Crouen spring (New Caledonia). Dominant phyla (>1% in average) are indicated in bold in the right-hand legend. Proteobacterial classes are distinguished by different blue colors. Sections of the bar chart of CANBW and CANBS samples are labeled with the corresponding taxa abbreviation as defined in brackets in the legend. CANB is the source bathtub, CANP is the outlet of a pipe below the source and CANA is the small pool below CANP before its entry into the river. W and S mean Water and Sediment, respectively. Values are averages of duplicate samples.

Twenty-three different phyla were identified across the water and sediment samples collected along with the water outflow of La Crouen spring (Figure 2). Among them, 12 major phyla (each >1% in average, all samples considered) represented more than 90% of all the prokaryotic sequences: *Actinobacteria*, *Bacteroidetes*, *Ca.* Gracilibacteria (formerly called GN02/BD1-5), *Chloroflexi*, *Cyanobacteria*, *Deinococcus-Thermus*, *Euryarchaeota*, *Firmicutes*, *Nitrospirae*, *Planctomycetes*, *Proteobacteria,* and *Spirochaetes*. 

**Figure 3:**
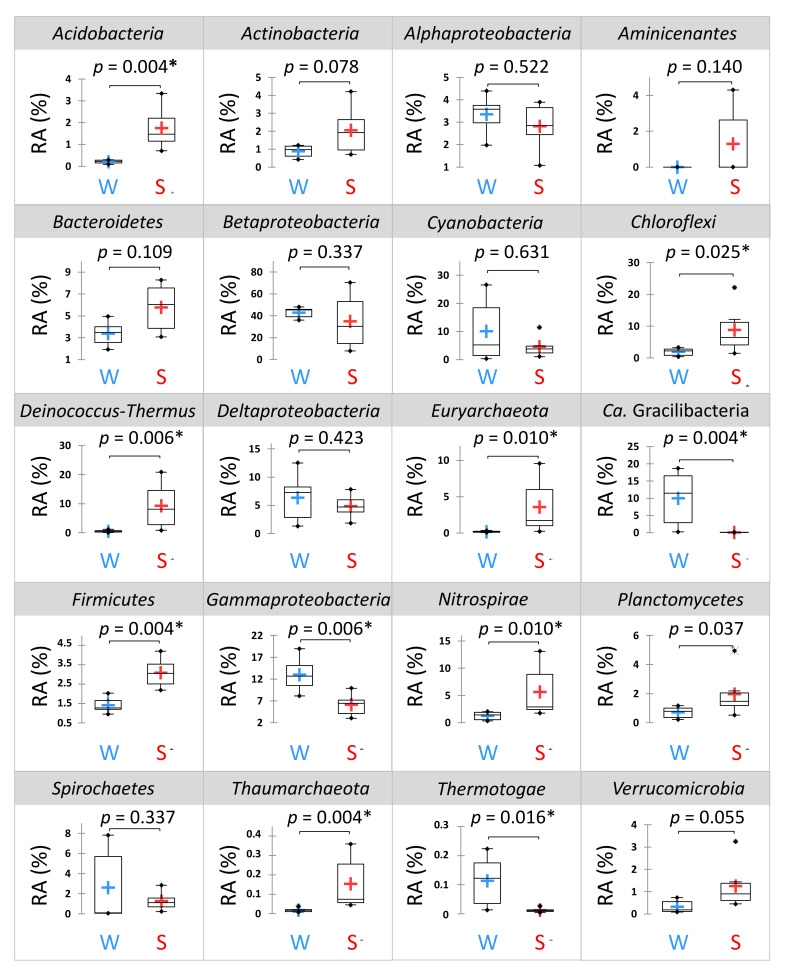
Boxplots showing variation of the relative abundance (RA; means ± standard deviations, *n* = 6) of the dominant phyla and proteobacterial classes (>1% in average) in the waters (W) and the sediments (S) of La Crouen Spring (New Caledonia). *p* values are obtained by Kruskal–Wallis test. *p* values < 0.05 indicate statistically significant differences between the two groups (W and S) and are indicated by asterisks.

*Proteobacteria* was predominant in all samples (57.1 ± 17.7%, 24.7−79.7%), mainly represented by *Betaproteobacteria* (38.9 ± 18.0%), except in CANBW (< 10%) (Figure 2). *Actinobacteria* (*p =* 0.004), *Chloroflexi* (*p =* 0.025), *Deinococcus-Thermus* (*p =* 0.006), *Euryarchaeota* (*p =* 0.01), *Firmicutes* (*p =* 0.004) and *Nitrospirae* (*p =* 0.01) were significantly more abundant in sediments than in waters (Figure 3). On the contrary, *Gammaproteobacteria* (*p =* 0.006), *Ca.* Gracilibacteria (*p =* 0.004) and *Thermotogae* (*p =* 0.016) were the only taxa (class/phyla) found in greater abundance in water than in sediment. 

### 3.4. Distribution and Diversity of Dominant Bacterial OTUs in La Crouen Spring

**Figure 4:**
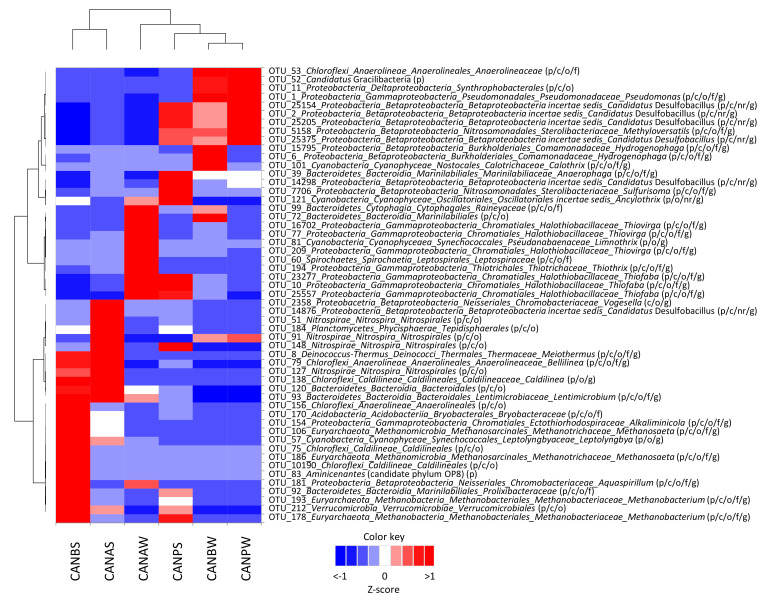
Heat map visualizing the Z-score distribution of the relative abundance of the dominant OTUs (>1% on average) and their respective taxonomic affiliations (from phylum to the less inclusive rank that can be attributed with certainty) in the waters and sediment samples along the water outflow of La Crouen spring (New Caledonia). The taxonomic ranks related to OTUs are in brackets. The letters p, c, o, f, g, and nr correspond to phylum, class, order, family, genus, and no rank, respectively. Abundance data used are averages of duplicate samples per sites. CANB is the source bathtub, CANP is the outlet of a pipe below the source and CANA is the small pool below CANP before its entry into the river. W and S mean Water and Sediment, respectively. Details on taxonomic affiliation of each dominant OTUs, with identity percentage with closest cultivated strains, are given in Tables S2 and S3.

Forty-five bacterial OTUs were found in significant amounts (> 1% of prokaryotes) in La Crouen sediment and water samples (Tables S2 and S3). On average, they represented 59.3 ± 8% and 72.3 ± 4.5% of prokaryotes in sediments and waters, respectively. Their relative abundance varied along with the water outflow and the associated sediments (i.e., collected at the same point) (Figure 4). Ten proteobacterial OTUs were abundant in the waters (CANBW) but rare in the CANB sediments. Dominant betaproteobacterial OTUs in waters were mainly affiliated with: (i) potential sulfur-reducing, incomplete denitrifiers *Ca.* Desulfobacillus denitrificans (OTUs #2, #25154 and #25205; ~20% of water prokaryotes), (ii) H_2_-oxidizing *Hydrogenophaga* (OTUs #6 and #15795, >5% in CANBW, rare in other samples), and (iii) methylotrophic *Methyloversatilis* (OTU #5158, 4.5% in CANBW). *Gammaproteobacteria* OTUs belonging to the sulfur-oxidizers genera *Thiofaba* (OTUs #10, #23277, and #25557) and *Thiovirga* (#77 and #16702) represented more than a third of the prokaryotes in oxic CANAW samples. The sole dominant OTU belonging to *Deltaproteobacteria* (OTU #11) was exclusively found in waters (Table S2).

The other most abundant non-proteobacterial OTUs (*n* = 24) were less abundant than the proteobacterial ones in the waters CANBW, except for 4 OTUs exclusively found in noticeable amounts in waters samples: (i) OTU #52 affiliated to the candidate phylum *Gracilibacteria* (> 10% of the water prokaryotes), (ii) OTUs #72 and #99 affiliated to Bacteroidetes and (iii) the cyanobacterial OTU #101 related to the genus *Calothrix* (Table S3). *Leptolyngbya* OTU #57 (*Cyanobacteria*) was abundant in the sediments CANBS and CANAS. The *Meiothermus* OTU #8 (*Deinococcus-Thermus*) was also abundant in these sediment samples (> 10% of the prokaryotes) but was < 1% in the water CANBW. The *Anaerophaga* OTU #39 (*Bacteroidetes*) represented 1.3% of reads in CANBW and up to 5.6% in CANPS but was rare (0.1%) in CANAW. Differences in dominant *Chloroflexi* OTUs were also observed among CANB samples since *Anaerolineaceae* OTU #53 dominated in water and *Caldilineaceae* in sediment. 

### 3.5. Diversity of Dominant Archaeal OTUs in La Crouen Spring

Four archaeal OTUs were detected in sediment samples at significant abundances (> 1% of the prokaryotic sequences) but not in water samples (Table S3). They were mainly found in the sample CANBS and were affiliated with the genera: (i) *Methanosaeta* (OTUs #106 and #186, 4.5% of prokaryotes, >96% identity) and (ii) *Methanobacterium* (OTUs #178 and #193, 4.5% of prokaryotes, >99% identity) both composed of methanogenic members. On the contrary, the sum of archaeal members represented less than 0.5% of total prokaryotes in water samples (Figure 2). The most abundant OTUs (each accounting for ~0.02% of prokaryotes and >5% of *Archaea*) were distantly related to cultivated *Archaea* but closely related (>99% identity) to two sequences previously detected in the peridotite-hosted PBHF and assigned to the candidate phylum *Hadesarchaea* (KJ149166) or *Methanosarcinales* LCMS (KJ149165) [10].

### 3.6. Cultivation and Isolation of Alkaliphilic Methanogens from La Crouen

**Figure 5:**
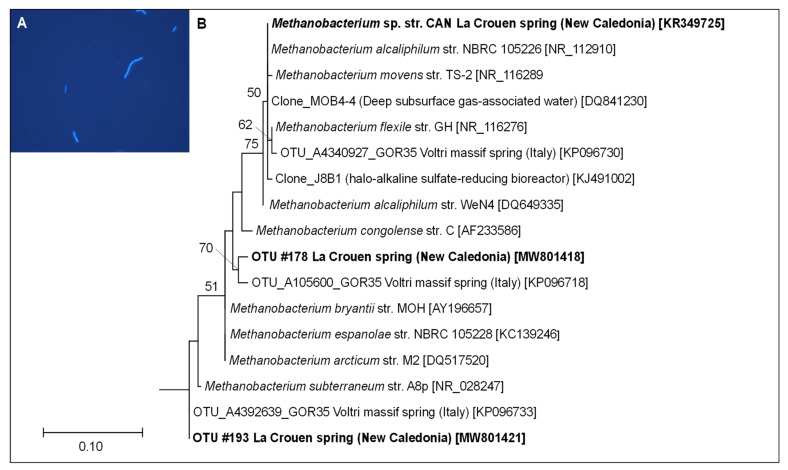
Micrograph of autofluorescent cells of strain CAN (**A**) and maximum likelihood (ML) phylogenetic tree of 16S rRNA gene sequences showing the position of strain CAN among its closest neighbors belonging to the genus *Methanobacterium* and the closest environmental sequences retrieved from La Crouen spring (this study) (**B**). The sequences obtained in this study are in bold. The closest environmental sequences retrieved from a BLAST analysis as well as the serpentinizing Voltri site were added. All positions containing gaps and missing data were eliminated. There was a total of 223 positions in the final dataset. *Methanopyrus kandleri* was used as outgroup (not shown). Bootstrap values higher than 50% (based on 1000 replicates) are shown at branch nodes. Accession numbers are indicated in parentheses. Bar: 0.1 substitutions per 100 nucleotides.

Anaerobic enrichment cultures from CANB sediment amended with methanogenic substrates were performed at an initial pH value of 9 to target alkaliphilic methanogens. After two weeks of incubation at 37 °C, CH_4_ production was detected in all enrichment cultures, accompanied by a decrease in H_2_. No CH_4_ production was observed in the non-inoculated cultures used as controls. The highest dilution of CANB (10^−4^) cultures, yielding a significant CH_4_ production, was transferred into a fresh culture medium with H_2_/CO_2_ as sole energy and carbon sources. It displayed a high CH_4_ production coupled to a total H_2_ consumption after 15 days. Under the light of a microscope, the cultures appeared as non-motile strait rods (about 3–4 µm × 0.7 µm) showing a blue autofluorescence (with a 420 nm wavelength filter) under the UV light, characteristic of methanogenic cells (Figure 5A).

**Figure 6:**
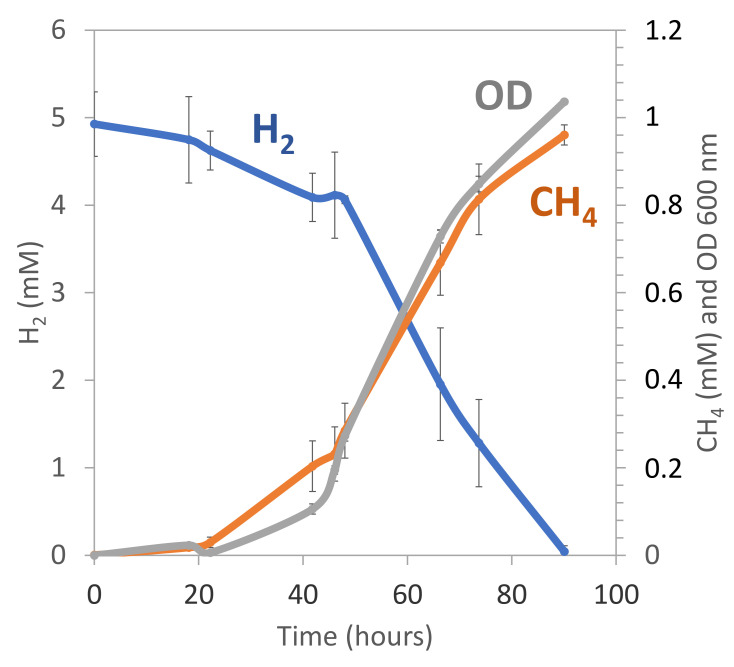
Kinetics of growth (optical density OD 600 nm × 10), hydrogen (H_2_) and methane (CH_4_) production of *Methanobacterium* sp. strain CAN. Values are means ± standard deviations of triplicated cultures.

One of these cultures was further purified by the three successive serial dilutions until it was deemed pure, as checked by microscopic observations and sequencing of its 16S rRNA gene. The corresponding phylogenetic analyses (based on ML method) indicated that the CAN strain belongs to the *Methanobacterium* genus and is closely related to *M. alcaliphilum*, previously isolated from Egyptian alkaline lakes sediments [52] (Figure 5B). The doubling time of CAN cells was four days at an optimal pH value of 9, during which H_2_ in the culture tube headspace was completely consumed to produce CH_4_ (Figure 6). The strain is a strictly hydrogenotrophic methanogen, able to use CO_2_ as carbon source, but unable to use alternatives substrates, such as acetate, formate, or methylated compounds (e.g., methanol).

## 4. Discussion

Methanogenic *Archaea* were dominant members of the sediments collected at La Crouen spring. Both *Methanobacteriales* and *Methanosarcinales* represented more than 5% of total prokaryotes and up to two-thirds of the *Archaea* community from the CANB samples. Their abundance decreases with increasing dissolved O_2_ along with the water outflow and accounts for less than 0.5% of prokaryotes in the water samples, explaining why we did not detect any dissolved CH_4_. The dominant *Methanosarcinales* OTUs (#106 and #186) were closely related (96−98% identity) to the acetoclastic species *Methanosaeta harundinacea* [53] and *Methanosaeta pelagica* [54]. Both OTUs were only distantly related to the sole archaeal phylotype Ced_A01 (KC578884, displaying 88% identity with *M. harundinacea*) detected at the hyperalkaline (pH~11.5), H_2_- and CH_4_-rich serpentinizing environment of The Cedars [55,56] and *Methanosarcinales* phylotypes from PBHF [10,11]. The *Methanobacterium* OTUs abundant in La Crouen sediments (OTUs #178 and #193) are closely related (99% identity) to hydrogenotrophic *Methanobacterium subterraneum* [57] and *Methanobacterium oryzae* [58]. Both *Methanobacterium* OTUs are closely affiliated with the dominant archaeal OTUs identified in the Voltri Ophiolitic spring GOR35 (pH~11.6), where dissolved H_2_ concentrations are low and CH_4_ moderate [59,60], a situation thus contrasting with that found for La Crouen waters.

*Methanobacterium*-like sequences were often detected in the hyperalkaline waters or sediments/carbonates of several on-land serpentinite-hosted ecosystems, such as the hyperalkaline springs of several ophiolites: Del Puerto (CA, USA) [61], Zambales (Philippines) [62], Voltri (Italy) [27,59], and Samail (Oman) [63,64]. They have also been reported at depth in rock formations of the continental crust [65,66]. *Methanobacterium* genus is composed, almost exclusively, of strict hydrogenotrophic members, i.e., using only H_2_ as an energy source [52,67]. Thus, the systematic detection and prevalence of the *Methanobacterium* phylotypes in continental alkaline springs strongly suggests that hydrogenotrophic methanogenesis constitutes the primary biological CH_4_ production process under alkaline conditions (pH > 9) [68]. At La Crouen spring, our cultivation experiments confirmed the presence of hydrogenotrophic methanogens. However, sampling spaced out over time would be necessary to evaluate better the CH_4_ production and its temporal fluctuation in both water and sediment, which could explain why we did not detect it at the time of sampling while it was previously reported [3].

This study describes a new alkaliphilic and hydrogenotrophic isolate (named CAN) closely affiliated (96−98% identity) with the hydrogenotrophic *Methanobacterium alcaliphilum, Methanobacterium flexile,* and *Methanobacterium movens*, isolated from the sediments of low-to-moderately saline (<35 g/L) and alkaline (pH 8.3−9.3) Egyptian and Qinghai-Tibetan Plateau lakes [52,67]. To our knowledge, this is the first time that an archaeon, a methanogen, was isolated from a possibly serpentinization-influenced site. Indeed, members of the genus *Methanobacterium* have been previously reported in enrichment cultures from the H_2_ and CH_4_-rich fluids of the NSHQ4 well in the Samail Ophiolite [64,69], but no axenic strain has been yet described from these cultures. Strain CAN could thus serve as a model organism for investigating CH_4_ production in serpentinizing environments, such as constraining the carbon and hydrogen isotope compositions of CH_4_ in these environments, as proposed by Miller et al. [69].

The candidate phylum ‘*Gracilibacteria’* (represented by the predominant OTU #52) was exclusively found in La Crouen water samples (> 10% of the water prokaryotes). *Ca.* Gracilibacteria members (formerly known as GN02/BD1-5) have been previously detected in diverse extreme or CH_4_-rich environments (e.g., hypersaline microbial mat, deep-sea hydrothermal vents, thermal springs) [70,71,72,73,74,75,76]. In field and groundwater CH_4_-spiked mesocosms studies, the growth of *Ca.* Gracilibacteria appeared to be stimulated by a period of CH_4_ starvation (after CH_4_ injection was stopped), suggesting they could benefit from the disappearance and death of other CH_4_-dependent populations [77,78]. Genomic and metagenomic analyses of *Ca.* Gracilibacteria have shown unusually limited metabolisms and potential syntrophic or parasitic lifestyles based on bacterial host-derived compounds and/or cell detritus [75,79,80]. In our study, abundant Ca. Gracilibacteria (OTU #52) co-exists with *Methyloversatilis* spp. (OTU #5158), which have been noted as the most persistent methylotrophs after many weeks of CH_4_ starvation in groundwater mesocosms [78]. This observation is consistent with the CH_4_ below the detection limit in our samples, even though it was previously detected at La Crouen spring [3]. Together with the presence of cultivated *Methanobacterium* sp., it may reflect temporal variation in biological CH_4_ production and consumption, perhaps linked to fluctuating H_2_ (abiotic or biological), feeding hydrogenotrophic methanogens.

The high proportion (~5%) of betaproteobacteria (OTUs #6 and #15795) in water sample CANBW depleted in H_2_ seems paradoxical. Indeed, these OTUs belong to the very closely related H_2_-oxidizing genera *Hydrogenophaga* and *‘Serpentinomonas’* [81]. The latter genus represents the most hyperalkaline known species firstly described from a serpentinizing environment (The Cedars, California) [81]. Indeed, *Hydrogenophaga*- or *Serpentinomonas*-like sequences have been abundantly detected in many continental hyperalkaline springs linked to the serpentinization of ultrabasic rock formations, such as The Tablelands Ophiolite (Newfoundland) [82], The Cedars Springs (California) [55,81], Cabeço de Vide Aquifer (CVA, Portugal) [83], Voltri Ophiolite (Italy) [59], or the PBHF samples (New Caledonia) [11,18]. Like in La Crouen, the H_2_ concentration in CVA and Voltri hyperalkaline waters was low or even below the detection limit [60,83,84]. The low concentration of dissolved H_2_ in these spring waters is most likely due to the high rate of H_2_ consumption by the hyperalkaliphilic H_2_-oxidizing bacteria.

Other betaproteobacterial OTUs found in large amounts in La Crouen waters are potentially involved in the sulfur and nitrogen cycles. They are represented mainly by the potential sulfate-reducing candidate genus ‘Desulfobacillus’ previously identified in acidic environments (e.g., geothermal system, acid mine drainage) [85,86]. A recent metagenomic analysis of a bacterial community performing anaerobic ammonium oxidation (anammox) has shown that a *Ca.* Desulfobacillus denitrificans phylotype, closely related to La Crouen OTUs (#2, #25154, #25205, #25375, #14298; 97–99% identity), can perform partial denitrification with N_2_ production [87]. The large abundance of Ca. Desulfobacillus denitrificans detected in our samples may explain the low sulfate and the high N_2_ contents of La Crouen waters.

Dominant gammaproteobacterial OTUs (#10, #23277, #25557, #77 and #16702), representing up to 20% of sediment prokaryotes at La Crouen, are closely related to chemolithoautotrophic sulfur-oxidizing *Thiofaba tepidiphila* [88] and *Thiovirga sulfuroxydans* (94–97% identity) [89], respectively isolated from a hot spring and a waste-water biofilm. The sole dominant deltaproteobacterial OTU (#11) found in La Crouen waters is only distantly related to cultivated bacteria (*Desulfominile limimaris*, 85% identity) but is closer (~92%) to sequences found in crustal waters collected at depth in the flank of the Juan de Fuca ridge (Eastern Pacific) (DQ513101) [90], in deep groundwaters producing CH_4_ (AB924419) [91], or in the waters of an Algerian hot spring (MH394146) [92].

Photosynthetic *Cyanobacteria* represent between 5% and 25% of the water prokaryotes of La Crouen. *Calothrix* (OTU #101) represented 2.5% of the prokaryotes in the water (CANBW), while *Leptolyngbya* accounted for ~ 5% of prokaryotes in CANB sediments. *Calothrix* OTU #101 is closely related (>99 % identity) to two sequences detected from an alkaline (pH~9) thermal spring (FJ206790) [93], and from the H_2_-sustained geothermal ecosystem of the Yellowstone National Park (AY862011) [94]. *Leptolyngbya* phylotypes were also found in microbial mats of the hyperalkaline springs of the Del Puerto (USA) [61] and Voltri (Italy) ophiolites [59,95]. Members of this genus can perform both oxygenic and anoxygenic photosynthesis depending on light irradiance and sulfide concentrations [96].

Anaerobic and moderately thermophilic members of the phyla *Bacteroidetes* (*Anaerophaga*) and *Chloroflexi* (*Anaerolineaceae*) were abundantly found in the CANBW water sample and co-exist with the major phyla *Proteobacteria* and Ca. Gracilibacteria. Potential sulfate-reducers are affiliated with the genus *Thermodesulfovibrio* (phylum *Nitrospirae*) that was initially established after the description of several species isolated from hot springs [97,98,99] and more recently described in a deep alkaline aquifer in Russia [100] and in hyperalkaline waters of the Samail Ophiolite (Oman) [63]. As observed in the Samail waters, in the La Crouen spring, thermophilic anaerobes of the genus *Thermodesulfovibrio* seem to co-exist with aerobic thermophiles of the genus *Meiothermus*. This suggests that La Crouen spring waters result from a mixing of deep (hot and anoxic) and surface (cold and oxygenated) waters before surface discharge. *Meiothermus* members (formerly *Thermus*) have been isolated from various geothermal springs [101,102], and they were also detected in several springs discharging hyperalkaline H_2_-rich waters, such as those of the Samail Ophiolite (Oman) [63], the Zambales Ophiolite (Philippines) [62], and the PBHF (New Caledonia) [18]. Rempfert et al. [63] explained such aerobic bacteria in Oman anoxic waters either by their ability to perform anaerobic respiration or slow aerobic respiration in deep subsurface waters or O_2_ contamination by subsurface water infiltrations through rock fractures.

## 5. Conclusions

The prokaryotic communities inhabiting La Crouen spring show several similarities with other thermal springs worldwide. The predominance of potential sulfur-metabolizing prokaryotes (*Ca.* Desulfobacillus, *Thermodesulfovibrio, Thiofaba,* and *Thiovirga*) suggests that sulfur redox reactions play a key role in the sustainability of microbial communities in La Crouen spring. The abundance of microorganisms using H_2_ as an energy source, either in microaerophilic (e.g., *Hydrogenophaga*) or anaerobic (e.g., *Thermodesulfovibrio, Methanobacterium*) conditions, commonly detected in serpentinizing environments (e.g., PBHF, Voltri ophiolite) suggests a possible contribution of in-depth H_2_-rich hot fluids, generated by serpentinization reactions on subsurface peridotite rocks to the hydrothermal ecosystem of La Crouen spring. The origin of such fluids may be local (in La Crouen area) or further south in the Prony area, where the peridotite nappe outcrops on the surface. This hypothesis could notably explain the presence of thermophilic bacteria (*Thermodesulfovibrio*, *Meiothermus*) and archaea (*Ca.* Hadesarchaea) previously detected in PBHF [10] by microbial dispersion via water circulation within the New Caledonian Ophiolite. The abundance of H_2_-utilizing microorganisms, disregarding their precise taxonomic affiliation, which is not specific to the serpentinite-hosted environment, still is a good proxy of H_2_-rich environments. In La Crouen spring, like in other terrestrial alkaline springs, the different microbial compositions between water and sediment indicate that the oxygenation of the subsurface anoxic water is a critical parameter shaping the microbial community structure. Finally, a novel alkaliphilic methanogen, *Methanobacterium* sp. CAN, was isolated from La Crouen, demonstrating the existence of hydrogenotrophic methanogenesis in this ecosystem. However, its low abundance in the water samples where *Ca.* Gracilibacteria proliferates together with the methylotrophic bacteria *Methyloversatilis* suggests the existence of a CH_4_ cycle, rhythmed by the fluctuation of H_2_-rich water originating from the serpentinizing area, alimenting this ecosystem.

## Figures and Tables

**Table 1:** In situ physicochemical parameters, alpha diversity and gene abundances in sediment and water samples along the water outflow of La Crouen spring (New Caledonia). Microbial data values are means ± standard deviations of biological duplicates.

Samples ^1^	CANBW	CANPW	CANAW	CANBS	CANPS	CANAS
Types	Water	Water	Water	Sediment	Sediment	Sediment
pH ^2^	9.0–9.1	9.0–9.1	9.0–9.1	N/A ^3^	N/A ^3^	N/A ^3^
Temperature (°C) ^2^	41	41	40	N/A ^3^	N/A ^3^	N/A ^3^
O_2_ (mg/L) ^2^	0.7	5.3	4.9	N/A ^3^	N/A ^3^	N/A ^3^
ORP (mV; ref. Ag/AgCl) ^2^	−351	−333	−260	N/A ^3^	N/A ^3^	N/A ^3^
Shannon index (*H*)	4.60 ± 0.03	3.70 ± 0.02	4.16 ± 0.02	5.46 ± 0.06	4.45 ± 0.60	5.02 ± 0.19
Simpson index (*D*)	0.965 ± 0.001	0.928 ± 0.001	0.916 ± 0.007	0.974 ± 0.008	0.941 ± 0.021	0.962 ± 0.020
Bacterial 16S rDNA (copies/g or copies/L)	5.50 × 10^7^	3.04 × 10^7^	1.06 × 10^9^	6.97 × 10^9^	2.42 × 10^9^	6.02 × 10^9^
Archaeal 16S rDNA (copies/g or copies/L)	2.31 × 10^6^	1.92 × 10^6^	1.63 × 10^7^	5.38 × 10^7^	6.59 × 10^7^	8.69 × 10^7^
*mcrA* (copies/g or copies/L)	2.23 × 10^5^	1.17 × 10^5^	3.14 × 10^6^	1.95 × 10^7^	8.17 × 10^6^	2.19 × 10^7^

^1^ CANB is the source bathtub, CANP is the outlet of a pipe below the source and CANA is the small pool below CANP before its entry into the river. W and S mean Water and Sediment, respectively. ^2^ Physicochemical values have been measured in situ. ^3^ N/A: Not applicable.

## Data Availability

The 16S rRNA gene sequences obtained in this study have been deposited in the Genbank database under the accession numbers MW801388-MW801436 and KR349725 (for the CAN strain).

## Supplementary Materials

The following are available online at https://www.mdpi.com/article/10.3390/microorganisms9071360/s1, Table S1: Chemical composition of the bath water CANBW at La Crouen spring (New Caledonia); Table S2: Blast analysis on the dominant proteobacterial OTUs (>1% of total sequences in at least one sample) obtained from La Crouen spring; Table S3: Blast analysis on the dominant non-proteobacterial OTUs (>1% of total sequences in at least one sample) obtained from La Crouen springs.
