# Supplementary material for: Procaryotic Diversity and Hydrogenotrophic Methanogenesis in an Alkaline Spring (La Crouen, New Caledonia)
Source: Microorganisms. 2021 Jun 23;9(7):1360. doi: 10.3390/microorganisms9071360 (PMC8307142; doi:10.3390/microorganisms9071360)
Supplement: Supplementary file 1 [file microorganisms-09-01360-s001.zip › microorganisms-1244191-supplementary.pdf]

Table S1. Chemical composition of the bath water CANBW at La Crouen spring (New Caledonia).

Source	This work	Cox et al., 1982
Na (mM)	2.24	2.41
K (µM)	30	0.05
Ca (µM)	35	0.03
Mg (µM)	0.64	8.23
Sr (µM)	4.41	ND[3]
Ba (nM)	24	ND[3]
Si (mM)	0.81	1.00
DIC as Cinorg (mM)	1.36	0.34
NPOC as Corg (mM) [1]	0.05	ND[3]
Cl (mM)	0.24	0.34
SO_4 (mM)	0.16	0.15
NO_3 (µM)	0.44	ND[3]
F (µM)	23.63	ND[3]
PO_4 (µM)	0.30	ND[3]
N_2 (µM)	363	ND[3]
H_2 (µM)	BDL[2]	ND[3]
CH_4 (µM)	BDL[2]	ND[3]
O_2 (µM)	54	ND[3]
CO_2 (µM)	BD[2]	ND[3]

[1] NPOC: Non Purgeable Organic Carbon
[2] BDL: Below detection limit
[3] ND: Not determined

Table S2. Blast analysis on the dominant proteobacterial OTUs (>1% of total sequences in at least on sample) obtained from La Crouen spring.

**OTUs (Accession number)**	Closest cultivated relative retrieved from NCBI nucleotide database			% of sequences per samples											
	**Species (Accession number)**	**Taxonomy (Proteobacterial class, order, family)**	**Identity (%)**	CANBS1	CANBS2	CANBW1	CANBW2	CANPS1	CANPS2	CANPW1	CANPW2	CANAS1	CANAS2	CANAW1	CANAW2
OTU_181 (MW801419)	*Aquaspirillum serpens* (AB680863)	*Betaproteo.; Neisseriales; Chromobacteriaceae*	99	1.1	0.5	0.0	0.0	0.0	0.0	0.0	0.0	0.1	0.0	0.3	0.4
OTU_5158 (MW801426)	*Methyloversatilis* sp. DMSP-7 (KC860260)	*Betaproteo.; Nitrosomonadales ; Sterolibacteriaceae*	97	0.3	0.3	4.6	4.4	4.2	5.7	7.4	6.8	1.1	1.7	0.4	0.4
OTU_2 (MW801389)	*Ca. Desulfobacillus denitrificans* (AP021857)	*Betaproteo.; Candidatus Desulfobacillus*	99	0.8	0.7	9.9	10.1	12.5	13.6	16.0	18.1	3.0	4.2	1.1	1.2
OTU_25154 (MW801433)	*Ca. Desulfobacillus denitrificans* (AP021857)	*Betaproteo.; Candidatus Desulfobacillus*	97	0.4	0.4	5.4	5.4	6.4	7.7	8.9	10.0	1.7	2.5	0.5	0.5
OTU_25205 (MW801434)	*Ca. Desulfobacillus denitrificans* (AP021857)	*Betaproteo.; Candidatus Desulfobacillus*	97	0.2	0.1	2.3	2.1	2.3	3.2	3.5	3.6	0.7	1.0	0.3	0.2
OTU_25375 (MW801435)	*Ca. Desulfobacillus denitrificans* (AP021857)	*Betaproteo.; Candidatus Desulfobacillus*	97	0.0	0.0	0.7	0.8	0.9	1.0	1.3	1.6	0.2	0.4	0.1	0.1
OTU_14298 (MW801429)	*Ca. Desulfobacillus denitrificans* (AP021857)	*Betaproteo.; Candidatus Desulfobacillus*	97	0.0	0.0	0.2	0.4	2.1	1.3	0.4	0.4	0.2	0.2	0.1	0.1
OTU_7706 (MW801427)	*Sulfurisoma sediminicola* (NR_125471)	*Betaproteo.; Nitrosomonadales; Sterolibacteriaceae*	96	0.0	0.0	0.1	0.1	1.5	1.0	0.1	0.1	0.2	0.2	0.1	0.1
OTU_6 (MW801390)	*Hydrogenophaga atypica* (MG712818)	*Betaproteo.; Burkholderiales; Comamonadaceae*	99	0.2	0.2	4.2	3.3	0.3	0.3	0.2	0.2	0.4	0.3	0.4	0.4
OTU_15795 (MW801430)	*Hydrogenophaga aquatica* (NR_158120	*Betaproteo.; Burkholderiales ; Comamonadaceae*	99	0.1	0.0	2.4	1.8	0.0	0.0	0.0	0.0	0.1	0.1	0.1	0.1
OTU_2358 (MW801425)	*Vogesella indigofera* (MG011571)	*Betaproteo.; Neisseriales; Chromobacteriaceae*	95	0.4	0.1	0.1	0.1	0.5	0.3	0.1	0.1	6.9	3.4	0.2	0.2
**OTU_11** (MW801393)	*Desulfomonile limimaris* (NR_025079)	*Deltaproteo.; Syntrophobacterales; Syntrophaceae*	85	0.0	0.0	6.2	5.0	0.1	0.0	10.3	6.8	0.0	0.0	0.1	0.1
OTU_154 (MW801415)	*Alkalilimnicola ehrlichii* MLHE-1 (CP000453)	*Gammaproteo.; Chromatiales; Ectothiorhodospiraceae*	94	2.5	2.9	0.0	0.0	0.0	0.0	0.0	0.0	0.5	0.6	0.0	0.0
OTU_10 (MW801392)	*Thiofaba tepidiphila* (NR_041602)	*Gammaproteo.; Chromatiales; Halothiobacillaceae*	96	0.3	0.1	4.8	5.6	20.4	10.7	2.5	2.1	2.4	2.2	17.2	18.7
OTU_23277 (MW801432)	*Thiofaba tepidiphila* (NR_041602)	*Gammaproteo.; Chromatiales; Halothiobacillaceae*	94	0.1	0.0	1.3	1.5	6.4	2.9	0.7	0.6	0.7	0.7	5.2	5.2
OTU_25557 (MW801436)	*Thiofaba tepidiphila* (NR_041602)	*Gammaproteo.; Chromatiales; Halothiobacillaceae*	93	0.1	0.0	0.4	0.7	1.2	1.4	0.1	0.1	0.1	0.1	1.9	1.8
OTU_77 (MW801402)	*Thiovirga sulfuroxydans* (NR_040986)	*Gammaproteo.; Chromatiales; Halothiobacillaceae*	97	0.0	0.0	0.2	0.1	0.0	0.0	0.0	0.0	0.1	0.0	6.3	2.1
**OTU_16702** (MW801431)	*Thiovirga sulfuroxydans* (NR_040986)	*Gammaproteo.; Chromatiales; Halothiobacillaceae*	98	0.0	0.0	0.1	0.1	0.0	0.0	0.0	0.0	0.0	0.0	2.1	1.4
OTU_209 (MW801423)	*Thiovirga sulfuroxydans* (NR_040986)	*Gammaproteo.; Chromatiales; Halothiobacillaceae*	94	0.2	0.1	0.1	0.1	0.1	0.1	0.0	0.0	0.2	0.0	1.9	0.9
OTU_1 (MW801388)	*Pseudomonas putida* (MG846038)	*Gammaproteo.; Pseudomonadales; Pseudomonadacea e*	99	0.9	0.1	3.2	4.8	1.7	0.1	2.7	4.7	0.9	0.1	0.4	0.4
OTU_194 (MW801422)	*Thiothrix disciformis* (NR_118037)	*Gammaproteo.; Thiotrichales; Thiotrichaceae*	96	0.0	0.0	0.0	0.0	0.0	0.0	0.0	0.0	0.1	0.1	0.9	1.4

Table S3. Blast analysis on the dominant non-proteobacterial OTUs (>1% of total sequences in at least on sample) obtained from La Crouen springs.

**OTUs (Accession number)**	Closest cultivated relative retrieved from NCBI nucleotide database			% of sequences per samples											
	**Species (Accession number)**	**Taxonomy (Phylum, order, family)**	**Identity (%)**	CANBS1	CANBS2	CANBW 1	CANBW 2	CANPS1	CANPS2	CANPW1	CANPW2	CANAS1	CANAS2	CANAW 1	CANAW2
OTU_170 (MW801417)	*Paludibaculum fermentans* (NR_134120)	*Acidobacteria; Solibacterales; Bryobacteraceae*	92	0.9	1.1	0.0	0.0	0.0	0.1	0.0	0.0	0.1	0.2	0.0	0.0
OTU_93 (MW801406)	*Lentimicrobium saccharophilum* (MG264204)	*Bacteroidetes; Bacteroidales; Lentimicrobiaceae*	94	1.6	0.3	0.0	0.0	0.2	0.3	0.0	0.0	1.3	0.4	0.5	0.5
OTU_120 (MW801410)	*Lentimicrobium saccharophilum* (NR_149795)	*Bacteroidetes; Bacteroidales; Lentimicrobiaceae*	89	1.7	0.4	0.0	0.0	0.3	0.5	0.0	0.0	2.1	0.5	0.5	0.6
OTU_39 (MW801394)	*Anaerophaga* sp. (KX930003)	*Bacteroidetes; Marinilabiliales; Marinilabiliaceae*	99	0.2	0.1	1.4	1.1	5.6	3.4	1.7	1.3	1.3	1.0	0.1	0.1
**OTU_72** (MW801400)	*Prolixibacter bellariivorans* (LC015091)	*Bacteroidetes; Marinilabiliales; Prolixibacteraceae*	86	0.0	0.0	0.5	2.9	0.0	0.0	0.0	0.0	0.0	0.0	1.7	2.5
OTU_92 (MW801405)	*Sunxiuqinia faeciviva* (NR_108114)	*Bacteroidetes; Marinilabiliales; Prolixibacteraceae*	92	3.9	1.2	0.0	0.0	0.6	1.0	0.0	0.0	0.5	0.2	0.0	0.1
OTU_99 (MW801407)	*Raineya orbicola* (MF125288)	*Bacteroidetes; Cytophagales; Raineyaaceae*	89	0.0	0.0	0.6	1.2	0.4	0.0	0.0	0.0	0.1	0.1	1.9	2.5
OTU_79 (MW801403)	*Bellilinea caldifistulae* (NR_041354)	*Chloroflexi; Anaerolineales; Anaerolineaceae*	98	1.3	2.2	0.0	0.0	0.5	0.6	0.0	0.0	1.7	3.4	0.0	0.0
**OTU_53** (MW801397)	*Leptolinea tardivitalis* (NR_040971)	*Chloroflexi; Anaerolineales; Anaerolineaceae*	90	0.1	0.1	2.1	1.2	0.3	0.2	1.8	2.4	0.3	0.1	0.0	0.0
OTU_156 (MW801416)	*Thermomarinilinea lacunifontana* (NR_132293)	*Chloroflexi; Anaerolineales; Anaerolineaceae*	89	0.8	2.2	0.0	0.0	0.0	0.1	0.0	0.0	0.1	0.2	0.0	0.0
OTU_138 (MW801413)	*Caldilinea tarbellica* (NR_117797)	*Chloroflexi; Caldilineales; Caldilineaceae*	94	0.3	1.7	0.0	0.0	0.0	0.0	0.0	0.0	0.4	1.6	0.0	0.0
OTU_75 (MW801401)	*Litorilinea aerophila* (NR_132330)	*Chloroflexi; Caldilineales; Caldilineaceae*	89	3.0	3.9	0.0	0.0	0.0	0.0	0.0	0.0	0.0	0.0	0.0	0.0
OTU_10190 (MW801428)	*Litorilinea aerophila* (NR_132330)	*Chloroflexi; Caldilineales; Caldilineaceae*	87	2.0	1.9	0.0	0.0	0.0	0.0	0.0	0.0	0.0	0.0	0.0	0.0
**OTU_101** (MW801408)	*Calothrix* sp. (MT350509)	*Cyanobacteria; Nostocales; Calotrichaceae*	94	0.0	0.0	2.1	3.0	0.0	0.0	0.0	0.0	0.0	0.0	0.0	0.0
OTU_121 (MW801411)	*Ancylothrix terrestris* (KT819202)	*Cyanobacteria; Oscillatoriales; Phormidiaceae*	98	0.7	0.2	0.0	0.0	0.5	2.1	0.0	0.0	0.1	0.1	0.3	0.9
OTU_57 (MW801398)	*Leptolyngbya* sp. (MF405393)	*Cyanobacteria; Synechococcales; Leptolyngbyaceae*	98	8.7	2.0	0.2	0.2	0.0	0.0	0.0	0.0	2.6	1.2	0.5	0.7
OTU_8 (MW801391)	*Meiothermus ruber* (KC252987)	*Deinococcus-Thermus; Thermales; Thermaceae*	98	5.6	14.9	0.3	1.0	0.6	0.9	0.0	0.0	9.0	19.8	0.2	0.2
OTU_83 (MW801404)	*Candidatus Aminicenantes* (MK067121)	*Aminicenantes* (candidate phylum OP8)	100	3.5	4.3	0.0	0.0	0.0	0.0	0.0	0.0	0.0	0.0	0.0	0.0
**OTU_52** (MW801396)	*Candidatus Gracilibacteria* (CP042461)	*Candidatus Gracilibacteria*	92	0.0	0.0	12.0	9.9	**0.0**	0.0	17.7	17.1	0.0	0.0	0.2	0.1
OTU_127 (MW801412)	*Thermodesulfovibrio yellowstonii* (NR_074345)	*Nitrospirae; Nitrospirales; Nitrospiraceae*	89	1.2	0.5	0.0	0.0	0.0	0.1	0.0	0.0	1.5	2.0	0.0	0.0
OTU_148 (MW801414)	*Thermodesulfovibrio hydrogeniphilus* (NR_044075)	*Nitrospirae; Nitrospirales; Nitrospiraceae*	87	0.3	0.2	0.0	0.0	0.9	0.8	0.0	0.0	1.0	0.9	0.1	0.1
OTU_51 (MW801395)	*Thermodesulfovibrio yellowstonii* (NR_074345)	*Nitrospirae; Nitrospirales; Nitrospiraceae*	89	0.5	0.6	0.0	0.0	0.2	0.7	0.0	0.0	5.0	7.0	0.0	0.0
OTU_60 (MW801399)	*Leptospira alexanderi* (JQ988836)	*Spirochaetes; Leptospirales; Leptospiraceae*	90	0.1	0.0	0.0	0.0	0.0	0.0	0.0	0.0	0.7	0.1	6.5	6.7

OTU_212 (MW801424)	*Pedosphaera parvula* (AY960777)	*Verrucomicrobia; Verrucomicrobiales*	86	1.1	0.4	0.0	0.0	0.4	0.2	0.0	0.0	0.4	0.2	0.0	0.0
OTU_193 (MW801421)	*Methanobacterium subterraneum* (CP017768)	*Euryarchaeota; Methanobacteria; Methanobacteriales; Methanobacteriaceae*	99	0.5	1.3	0.0	0.0	0.0	0.3	0.0	0.0	0.0	0.1	0.0	0.0
OTU_178 (MW801418)	*Methanobacterium oryzae* (NR_028171)	*Euryarchaeota; Methanobacteria; Methanobacteriales; Methanobacteriaceae*	99	0.2	1.1	0.0	0.0	0.0	0.9	0.0	0.0	0.0	0.2	0.0	0.0
OTU_186 (MW801420)	*Methanosaeta pelagica* (NR_113571)	*Euryarchaeota; Methanomicrobia; Methanosarcinales; Methanosaetaceae*	96	2.0	1.0	0.0	0.0	0.0	0.0	0.0	0.0	0.0	0.0	0.0	0.0
OTU_106 (MW801409)	*Methanosaeta harundinacea* (KP231498)	*Euryarchaeota; Methanomicrobia; Methanosarcinales; Methanosaetaceae*	98	4.2	1.9	0.0	0.0	0.0	0.0	0.0	0.0	0.5	0.7	0.0	0.0
